# Parent-Reported Behavioral and Psychiatric Problems Mediate the Relationship between Sleep-Disordered Breathing and Cognitive Deficits in School-Aged Children

**DOI:** 10.3389/fneur.2017.00410

**Published:** 2017-08-11

**Authors:** Dale L. Smith, David Gozal, Scott J. Hunter, Leila Kheirandish-Gozal

**Affiliations:** ^1^Public Health Sciences, Pritzker School of Medicine, Biological Sciences Division, The University of Chicago, Chicago, IL, United States; ^2^Olivet Nazarene University, Bourbonnais, IL, United States; ^3^Department of Pediatrics, Pritzker School of Medicine, Biological Sciences Division, The University of Chicago, Chicago, IL, United States; ^4^Department of Psychiatry and Behavioral Neuroscience, Pritzker School of Medicine, Biological Sciences Division, The University of Chicago, Chicago, IL, United States

**Keywords:** sleep-disordered breathing, sleep apnea, snoring, cognition, attention deficit hyperactivity disorder, behavior problems, mediation

## Abstract

**Background:**

Numerous studies over the past several decades have illustrated that children who suffer from sleep-disordered breathing (SDB) are at greater risk for cognitive, behavioral, and psychiatric problems. Although behavioral problems have been proposed as a potential mediator between SDB and cognitive functioning, these relationships have not been critically examined.

**Methods:**

This analysis is based on a community-based cohort of 1,115 children who underwent overnight polysomnography, and cognitive and behavioral phenotyping. Structural model of the relationships between SDB, behavior, and cognition, and two recently developed mediation approaches based on propensity score weighting and resampling were used to assess the mediational role of parent-reported behavior and psychiatric problems in the relationship between SDB and cognitive functioning. Multiple models utilizing two different SDB definitions further explored direct effects of SDB on cognition as well as indirect effects through behavioral pathology. All models were adjusted for age, sex, race, BMI *z*-score, and asthma status.

**Results:**

Indirect effects of SDB through behavior problems were significant in all mediation models, while direct effects of SDB on cognition were not. The findings were consistent across different mediation procedures and remained essentially unaltered when different criteria for SDB, behavior, and cognition were used.

**Conclusion:**

Potential effects of SDB on cognitive functioning appear to occur through behavioral problems that are detectable in this pediatric population. Thus, early attentional or behavioral pathology may be implicated in the cognitive functioning deficits associated with SDB, and may present an early morbidity-related susceptibility biomarker.

## Introduction

Sleep-disordered breathing (SDB) encompasses several conditions involving the presence of increased upper airway resistance during sleep. Habitual snoring (HS), the most frequent symptom indicative of underlying upper airway resistance, exhibits prevalence estimates in school-aged children that have ranged from 1.5 to 27.6% (1) with median estimates revolving around 10–12%. Obstructive sleep apnea (OSA), which is commonly viewed as the most severe form of SDB, involves upper airway obstruction during sleep that leads to changes in blood oxygen and carbon dioxide levels of varying duration and severity with or without an accompanying arousal (2). OSA is estimated to affect 1–5% of school-aged children (1, 3, 4). HS in the absence of any gas-exchange abnormalities or disrupted sleep (also termed primary snoring or PS) is traditionally viewed as comprising the lowest severity end of the SDB spectrum (1).

Over the past several decades, the presence of significant relationships between SDB and numerous behavioral and cognitive outcomes in children has been extensively investigated. Several recent reviews and meta-analyses have concluded that children with SDB are more likely to suffer from cognitive and behavioral problems, as well as poorer educational outcomes [e.g., Ref. (5–7)]. However, the existence of a large number of studies of varying sample sizes using different outcome measures has often resulted in inconsistent patterns and conclusions across the multitude of studies, often failing to demonstrate a dose–response relationship between SDB severity and severity of cognitive or behavioral problems [for review, see Ref. (6, 8)]. Though many prior studies relied on small clinical samples, our recent large-sample analyses using clinical and community-based cohorts have suggested that even children with PS have significantly greater cognitive and behavioral pathology than control children across numerous parent-rated domains (9, 10). These analyses further suggested that behavioral pathology may be more robustly observed than cognitive problems in children with OSA, and appears to peak among children with PS who would, therefore, not be diagnosed as having OSA (10). However, cognitive problems may be greatest among children with the most severe SDB (9). In addition, snoring severity, which is predicated on parental reports, may serve as a more effective predictor of these problematic outcomes in children than the polysomnographically derived apnea–hypopnea index [AHI; (11)], an objective measure that requires overnight testing.

Support for causal interactions between SDB and cognitive and behavioral outcomes beyond observational research demonstrating deficits in children with SDB is increasing. Such evidence includes a recent randomized clinical trial that included 464 children, which demonstrated significantly greater improvements in treated children than in control children across behavioral outcome measures and some cognitive measures after 7 months (12, 13). Furthermore, numerous longitudinal intervention studies have illustrated improvements in behavior and cognitive outcomes following SDB treatment [for review, see Ref. (14, 15)]. However, it should be also noted that several recent longitudinal studies with modest follow-up sample sizes have failed to find an association between resolution of SDB and psychological outcomes (5, 16), and suggesting that effects of SDB on cognition or behavior may be either tenuous, affect only a proportion of susceptible children, or may not always be reversible (17, 18).

A variety of factors could potentially mediate any effects of SDB on adverse outcomes, including susceptibility to intermittent hypoxia and sleep fragmentation during respiratory events, inflammation and oxidative stress, genetic or epigenetic factors, or environmental variables (5, 19–21). Two widely referenced theories have proposed that either excessive nighttime arousals and the resulting lack of quality of sleep, or repeated hypoxic events during sleep, can result in neuronal injury, serving as the primary potential mediators of cognitive and behavioral dysfunction (14, 19, 22). In addition, the possibility has recently been advanced that behavioral problems may potentially mediate the effects of SDB on cognition, primarily as a result of increased behavioral disruptions reducing the capacity to attend to vital environmental information and experiences that typically enhance cognitive functioning (5, 23). If the latter supposition is correct, cognition in early childhood among children with SDB should be comparable to peers, and changes in cognition following treatment of SDB should be less robust than concurrent behavioral changes. Both of these assumptions have recently gained credence as illustrated by recent findings in several studies [e.g., Ref. (12, 16, 24)]. However, few studies have methodically tested mediation effects in SDB research in children. Spruyt and Gozal (25, 26) used a series of recursive structural equation models (SEMs) to show the interdependency between cognition, SDB, and BMI. Furthermore, Beebe et al. (23) showed that behavioral and attentional problems may serve as mediators of educational outcomes, using a sequence of analyses based on evidence conceptually needed to establish mediation (27). To our knowledge, no prior study has examined behavioral mediators using cognitive functioning as an outcome, or used modern mediation-based approaches to estimate direct effects of SDB as well as indirect effects of SDB through any potential mediator on any outcome variable. Therefore, the current study aimed to examine the hypothesis that behavioral problems may mediate any potential effects of SDB status on cognition. To this effect, different classification criteria of SDB were used along with approaches that included standard SEM, ratio-of-mediator-probability-weight (RMPW) analyses and established resampling-based mediation (RBM) methods. Structural equation modeling has been widely used to assess nature of relationships between multiple variables for decades, though RBM and RMPW have only been recently developed. These newer approaches expand upon traditional path analysis and SEM and provide useful additional information while relying on fewer assumptions.

## Materials and Methods

### Participants

Between 2006 and 2016, 1,115 children were recruited from the Louisville and Chicago areas. Children from Louisville were recruited through collaboration with public schools, and children in Chicago were recruited through community announcements and distribution of materials across the University of Chicago medical center. All children were between 5 and 10 years of age, and most had reported some form of sleep pathology. Demographic characteristics of the sample are outlined in Table 1. Some participants had missing information on one or more of the cognitive measures used, most often due to time constraints in assessments or data entry, and were, therefore, excluded in certain analyses. Resulting sample sizes for each model are indicated in the descriptions below. This study was carried out in accordance with the recommendations of University of Louisville and University of Chicago medical centers’ Institutional Review Boards, both of which approved all data collection and procedural elements of this research. Written informed consent from all children and/or their parents was obtained in accordance with the Declaration of Helsinki.

**Table 1:** Cohort demographic characteristics (*N* = 1,116^a^).

Characteristic	M (SD) or *n* (%)^a^
Age	6.84 (0.86)
Sex (male %)	598 (54.86%)
Race (black %)^b^	329 (34.06%)
BMI *Z*-score^c^	0.75 (1.35)
Asthma (yes %)	170 (20.12%)
AHI^d^	2.69 (6.16)
Snoring status^e^	
*Never*	117 (13.07%)
*Rarely*	77 (8.60%)
*Occasionally*	135 (15.08%)
*Frequently*	180 (20.11%)
*Almost always*	386 (43.13%)

*^a^Some covariates contained missing values, so percentages may not reflect the entire sample*.

*^b^Due to very small representation of other racial groups, only black and white children were included in this analysis*.

*^c^BMI refers to body mass index, computed as weight (kg)/height (cm)^2^. *Z*-score computation here uses age-appropriate norms*.

*^d^AHI represents apnea–hypopnea index, as described in the manuscript. Log transformed AHI was used for analyses due to strong positive skew. AHI scores ranged from 0 to 77.56*.

*^e^Rarely represents an estimate of snoring one night per week, occasionally two nights per week, frequently three nights per week, and almost always four or more nights per week*.

### Behavioral Pathology Measures

Behavioral measures were chosen to obtain a wide coverage of behavioral pathology using a small number of clinically validated measures. These included the *Hyperactivity, Psychosomatic*, and *Inattention* subscales of the Conners’ Parent Rating Scales-Revised [CPRS-R; (28)], and the *Internalizing* and *Externalizing* index scores of the child behavior checklist-revised [CBCL; (29)]. Both scales utilize parent ratings to determine the occurrence and severity of a variety of problematic behaviors and psychiatric concerns in children. The *internalizing* score from the CBCL is a partial summary score that combines subscale scores from items measuring withdrawn, somatic complaints, and anxious-depressed symptomology. Similarly, the CBCL *externalizing* score reflects aggression and conduct problems subscale scores.

Both the CBCL and CPRS-R have shown acceptable psychometric properties upon examination. Estimates of internal consistency are strong for all subscales of the CPRS-R [0.77–0.93; (28)] and the CBCL [0.71–0.89; (30)]. Since both scales were designed as screens for clinical use, a wealth of research has examined, and generally supported, their use at detecting a variety of childhood conditions involving emotional or behavioral problems, including autism, disruptive behavior disorders, and bipolar disorder [e.g., Ref. (31–34)]. A recent meta-analysis has also suggested moderately strong pooled sensitivity of 0.75 and 0.77 and specificity of 0.75 and 0.73 for the CPRS-R and CBCL, respectively, for detecting attention deficit hyperactivity disorder in children and adolescents (35).

### Cognitive Functioning Measures

Intellectual functioning was assessed by both *verbal* and *non-verbal cluster* scores on the Differential Ability Scale [DAS; (36)]. We also included individual subtest scores from both the NEPSY and the NEPSY-II (37) to capture potential deficits in a variety of neurocognitive domains, including attention/executive-functioning, language ability, and visuospatial processing. These included the *design copying, phonological processing, speeded naming, arrows*, and *comprehension of instructions* subtests from the NEPSY-II, as well as the *visual attention* and *tower* subtests from the original NEPSY.

Estimates of internal reliability for the DAS are over 0.70 for all subtests, and estimates of inter-rater reliability are greater than 0.90 (36). In addition, clinical utility of overall cluster scores has been demonstrated (38, 39), and DAS performance is moderately-to-strongly associated with other intellectual functioning measures, such as the Kaufman and Wechsler instruments (38). Research has also supported the overall psychometric properties of the NEPSY, with internal consistency and reliability estimates ranging from 0.79 to 0.91, and inter-rater reliability estimates above 0.97 (40, 41). Subtests also relate well with relevant subtests of other commonly utilized measures, such as Wechsler intelligence tests and the Delis–Kaplan executive functions system, and the utility of the NEPSY in identifying childhood cognitive functioning problems with various clinical samples across several domains has also been supported (41).

### Sleep Measures

All children were assessed overnight through standard nocturnal polysomnogram (NPSG). Estimates for AHI were scored according to current American Association of Sleep Medicine guidelines by pediatric sleep experts, and refer to the number of sleep disruptions per hour of sleep. Scorers were blind to behavioral test results and, similarly, the developmental neuropsychologists performing the behavioral and cognitive battery assessments were unaware of the sleep study findings. Parent reports were utilized to obtain snoring information. Snoring status was reported by a parent as “never,” “rarely (once per week),” “occasionally (twice per week),” “frequently (three times per week),” and “almost always (more than four times per week)” as part of a validated and commonly used questionnaire that has demonstrated convergence with objective measures of snoring (25, 26, 42).

### Analytic Strategy

**Figure 1:**
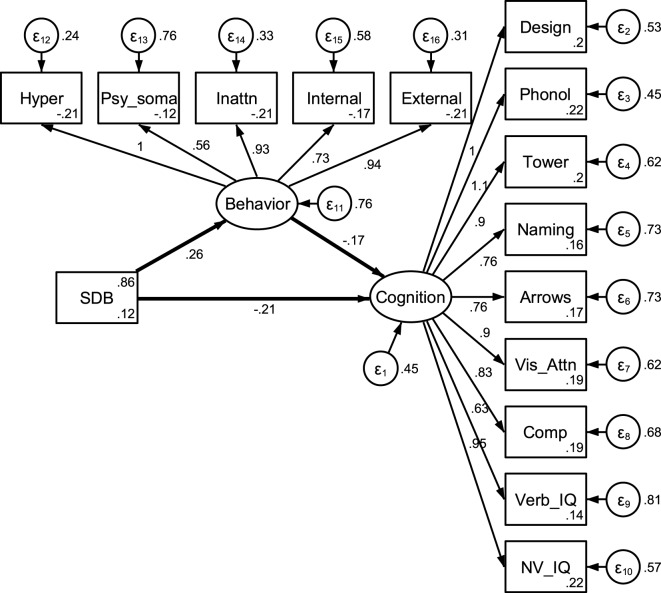
Unadjusted mediation model. Note: estimates were obtained using full information maximum likelihood and represent estimates prior to adjusting for demographic covariates. Behavioral variable abbreviations represent hyperactive, psychosomatic, and inattention CPRS subtest scores, and internalizing and externalizing CBCL subtest scores. Cognitive abbreviations represent design copying, phonological processing, tower, speed naming and arrows NEPSY subtests, and DAS verbal and non-verbal subtests.

Mediation models were examined using behavioral problems as a mediator of the relationship between SDB and cognitive functioning (see Figure 1). We adjusted for age, sex, BMI, race, and asthma status in both the outcome and mediation models for all analyses, as all are commonly found to be associated with both SDB status and behavioral or cognitive functioning in children [e.g., Ref. (5, 43)]. Due to some skewness in cognitive and behavioral measures, a Box-Cox transformation was utilized for all measures (44). Initial exploration also indicated that relationships between SDB and both behavior and cognition overall domain scores were significant at *p* < 0.05.^1^ To reflect empirical and clinical differences in the assessment of SDB and to determine whether differences in mediation exist based on changes in these characterizations, SDB status was dichotomized in two different ways in separate analyses. A description and explanation of each follows.

*The first characterization of SDB* compared SDB of any type (*n* = 871) to controls (*n* = 145). A child who snored “occasionally” or more frequently *or* had an AHI >1/h TST was determined to suffer from SDB. Although some debate exists regarding the use of the AHI of 1/h TST, this cut off is commonly used in research and clinical practice [e.g., Ref. (45)]. This characterization allowed for a comparison of control children who do not snore regularly with children who suffer from SDB and ignored any severity or classification variations within those who suffer from SDB, although AHI was incorporated into this categorization.

*The second characterization* defined SDB purely based on snoring status and attempted to compare children who snore regularly (*n* = 701) with those who do not (*n* = 194). Regular snoring was defined as “occasionally” or more frequent. AHI was ignored in this characterization, based on prior research that suggested that snoring status serves as a more effective predictor of cognitive and behavioral pathology (11). Furthermore, the correlation between snoring status and AHI was significant but not particularly strong in this sample (*r*_s_ = 0.24, *p* < 0.01), suggesting that this characterization could meaningfully differ from the first in identification of pathology and associations with outcomes.

Three analytic methods were utilized for mediation analysis to ensure robustness of the findings under slightly different procedures and identification assumptions. All three approaches attempted to test the theory that behavioral and psychiatric problems mediate the relationship between SDB and cognitive functioning problems. These mediation analytic methods are capable of assessing direct effects of SDB status on cognitive functioning, as well as indirect effects of SDB status on cognition through behavioral and psychiatric problems. Determining direct and indirect effects is typically accomplished through fitting two linear models. The first model predicts the outcome using an exposure or treatment variable, a mediator, and any relevant covariates. The second predicts the mediator using the exposure variable and any relevant covariates. Direct and indirect effects of an exposure variable on an outcome are traditionally estimated using coefficients obtained through these models, as discussed in reviews elsewhere [e.g., Ref. (46, 47)]. However, unlike typical SEM-based analyses, the resampling approach (RBM) and RMPW methods utilized here are also capable of assessing SDB-by-mediator interactions. Such interactions can determine whether any effect of behavioral pathology on cognitive functioning differs depending on child’s SDB status, which is both novel to this field and of potential interest in this analysis.

Our first analytic method utilized a traditional SEM-based approach to examine a latent behavioral problems variable as a mediator of the relationship between SDB and cognitive functioning. This approach involves assessing the potential structural relationships between both measured variables and latent variables. In our current work, cognition and behavior problems, which are assessed through various measurements, are hypothesized to comprise aspects of the underlying constructs they represent. This approach allowed for examination of direct and indirect effects of SDB on a latent cognition variable while assessing loadings of measured variables onto the latent behavior and cognition domains, as well as overall fit of the structural model. All five behavioral measures were included as measured variables for the latent behavioral problems domain, and all nine cognitive measures were included for the cognitive functioning domain. Full information maximum likelihood estimation (FIML) was utilized for SEM estimation due to the presence of some missing values.^2^

The second analytic method utilized a resampling-based mediation (RBM) approach introduced by Imai and colleagues (47–50). This approach generates linear models for the outcome and mediator as outlined above, followed by repeated simulations estimating potential values of both models and appropriate levels of statistical uncertainty through bootstrapping or Monte Carlo approximations. This is a flexible approach that easily extends to non-parametric estimation and sensitivity analysis. We utilized 100 simulations and determined SEs for significance testing using bootstrapping with at least 1,000 resamples for all mediation analyses using this approach. Additional benefits of this approach include the ability to examine SDB-by-mediator interactions and the existence of a readily available method of conducting sensitivity analyses using existing software.

Our third analytic mediation approach used RMPWs (51, 52). This involved first fitting propensity-score based inverse-probability-of-treatment weights in an attempt to balance the covariates noted above across SDB groups. Ratio-of-mediator-probability weights were then generated based on conditional probabilities of mediator values under alternative SDB conditions. Weighted analyses were next conducted for the mediator and outcome models to assess any direct or indirect effects of SDB, as well as SDB–mediator interaction. Due to some remaining imbalance in covariates across SDB groups, we further adjusted for age, sex, BMI, race, and asthma status in the outcome model that generated direct and indirect effects estimates. At least 1,000 bootstrapped samples were utilized to determine SEs for significance testing for all RMPW analyses. This new propensity score-based approach may rely on fewer model assumptions than the SEM or resampling approach (46).

Multiple methods were chosen for this analysis to ensure robustness of conclusions despite differing assumptions inherent to each analytic method described above [for review see Ref. (46)]. Both RBM and RMPW are based on the counterfactual (potential outcomes) framework for causal inference, which has been effectively described elsewhere [e.g., Ref. (46, 48, 53)]. To complete RBM and RMPW analyses, which unlike SEM do not traditionally utilize latent variables, factor scores were created to obtain individual scores for overall behavioral problems and overall cognitive functioning. These scores were created using maximum likelihood estimation (FIML), and included all five behavioral outcomes and nine cognitive outcomes.^3^ All SEM models utilized 1,097 participants due to the ability of FIML methods to accommodate missing data. Both RBM and RMPW analyses utilized sample sizes of 623 participants. All analyses were conducted using Stata version 14 and R statistical software.

## Results

As expected, estimates of the relationship between SDB status and parent-rated behavior and psychiatric problems were significant in all analyses that follow (*p*s < 0.01), children with SDB experiencing greater behavioral pathology. The association between reported behavioral problems and cognitive functioning was also significant and negative in all models (*p*s < 0.01), as greater behavioral pathology was associated with lower overall cognitive functioning. The five behavioral measures outlined above reflected behavioral problem severity and the nine cognitive measures comprised the latent cognitive functioning variable for SEM or creation of factor scores. Correlations between these behavioral measures ranged from 0.36 to 0.71, and all significantly loaded onto the latent behavioral problems domain for SEM or creation of factor scores (*p*s < 0.01). Similarly, correlations between cognitive measures ranged from 0.19 to 0.55, and all measured variables loaded onto the latent cognitive functioning domain (*p*s < 0.01).

### SEM Approach

Using an SDB cut-off value that compared children suffering from SDB (AHI >1 or snoring status at least occasional) with control children, indirect effects of SDB on cognitive functioning through behavior problems were significant (*p* < 0.01) though direct effects of SDB on cognition were not (*p* = 0.05; see Table 2). The same trend existed when characterizing SDB exclusively based on snoring status; the indirect effect of SDB through behavioral problems was significant (*p* < 0.01), but not the direct effect of SDB on cognition (*p* = 0.13). Both direct and indirect effects of SDB were negative, reflecting that children with SDB had lower overall cognitive functioning, though it appears that this effect is primarily mediated through behavior problems. Although the primary purpose for this analysis was examination of direct and indirect effects rather than construction of a comprehensive model of SDB effects, fit indices for the SEM model likely reflect adequate model fit (54, 55): snoring and AHI model: χ^2^(148) = 987.79, RMSEA = 0.07; snoring model: χ^2^(148) = 994.33, RMSEA = 0.07, though no modifications were made to the hypothesized model based on modification or fit indices.

**Table 2:** Estimates of direct and indirect effects of sleep-disordered breathing (SDB) on cognition.

Model^a^	Direct effect estimate (95% CI)	Indirect effect estimate (95% CI)	SDB × Beh. interaction estimate (95% CI)
**SDB 1 *(snoring and AHI)***			
SEM	−0.12 (−0.24, 0.01)	−0.05 (−0.09, −0.02)*	−
RBM^b^	−0.08 (−0.21, 0.05)	−0.04 (−0.08, −0.02)*	−0.01 (−0.05, 0.03)
RMPW	−0.05 (−0.18, 0.09)	−0.05 (−0.08, −0.02)*	<0.01 (−0.17, 0.17)
**SDB 2 *(snoring status)***			
SEM	−0.09 (−0.20, 0.02)	−0.07 (−0.10, −0.03)*	−
RBM	−0.09 (−0.20, 0.03)	−0.05 (−0.08, −0.03)*	0.01 (−0.03, 0.05)
RMPW	−0.07 (−0.19, 0.06)	−0.05 (−0.09, −0.02)*	<0.01 (−0.15, 0.15)

*^a^SDB characterization 1 reflects snoring at least “occasionally” or AHI >1; SDB characterization 2 reflects snoring at least “occasionally” without taking AHI into consideration*.

*^b^RBM approach confidence intervals are Quasi-Bayesian*.

*Reflects p < 0.05 after adjusting for age, sex, race, BMI, and asthma status in both mediation and outcome models.

*Parameter estimates are based on Box-Cox transformed values. SEM estimates utilize latent variables for behavior and cognition, RBM and RMPW approaches utilized factor scores. Values represent parameter estimates and 95% confidence intervals. Direct effects of SDB on behavior were significant for all models (*p*s < 0.01)*.

*SEM, structural equation model; RMPW, ratio-of-mediator-probability-weight; AHI, apnea-hypopnea index; RBM, resampling-based mediation*.

### RBM Approach

When characterizing SDB based on both snoring and AHI, average direct effects of SDB on cognitive functioning were again non-significant (*p* = 0.30) though average indirect effects of SDB through behavioral problems were significant (*p* < 0.01; see Table 2). Characterizing SDB exclusively based on snoring status also yielded a significant average indirect effect of SDB through behavioral problems (*p* < 0.01), though again the direct effect of SDB on cognition was not statistically significant (*p* = 14). Both direct and indirect effects were negative, reflecting that children with SDB had lower overall cognitive functioning, though much like the findings observed in the SEM analysis, these effects appeared to be primarily mediated by behavioral problems. No evidence of SDB by behavior interaction effects existed for either characterization of SDB (*p*s > 0.05), suggesting that any effects of behavior problems on cognitive functioning did not differ based on the child SDB status.

### RMPW Approach

Results using the RMPW approach were consistent with those reported using the SEM and RBM approaches. When characterizing SDB based on both snoring and AHI, the indirect effect of SDB on cognitive functioning through behavior was significant (*p* < 0.01) but the direct effect of SDB was not (*p* > 0.05). Similarly, when characterizing SDB exclusively relying on snoring status, the indirect effect of SDB through behavioral problems was significant (*p* < 0.01), but the direct effect of SDB on cognition was not (*p* > 0.05). As observed in the SEM and RBM approaches, both direct and indirect effects suggest that children with SDB had lower cognitive functioning. SDB by behavior interaction effects were again non-significant for both models (*p*s > 0.05), suggesting consistent effects of behavior on cognition across SDB status groups.

### Sensitivity Analysis

Causal interpretations of direct and indirect effects require that the *sequential ignorability assumption* for causal inference has been met. This assumption holds that, conditional measured covariates, the exposure and mediator variables can be viewed as randomized. This implies that no unmeasured confounding of the relationship between SDB and behavioral pathology, the relationship between behavioral pathology and cognitive functioning, or the relationship between SDB and cognitive functioning exists. This assumption is often very difficult to ascertain in observational and cross-sectional research studies, such that results regarding causation in such studies should always be interpreted with caution. To determine the plausibility of this assumption, sensitivity analyses were conducted for both characterizations of SDB using the procedure outlined by Imai and colleagues (48). This involved obtaining a sensitivity parameter (*r*) and determining the strength of a hypothesized unmeasured confounder or proportion of unexplained variance accounted for by said unmeasured confounders that would be needed to convert the average mediation effects to zero. Our results suggested that an unobserved confounder would need to account for approximately 3% of the overall variability in the mediator or outcome, or 4% of the unexplained variability, to fully negate our average mediation effect findings. Although such values cannot necessarily be interpreted in isolation, they likely indicate that the existence of a moderately strong unobserved confounder could potentially negate the effects observed here. Though this is certainly possible, it appears unlikely when considering that the combined effect of variables often cited as important, including *age, sex, BMI z-score, race*, and *asthma status* accounted for slightly under 3% of the total variance in behavioral functioning, and only *race* alone accounted for more than 2% of the variability in cognitive functioning.

## Discussion

In this study, our findings using multiple mediation analytical approaches that rely on differing identification assumptions suggest that parent-reported behavioral and psychiatric problems mediate the relationship between SDB status and cognitive functioning. Indirect effects of SDB through behavioral problems reliably predicted cognitive changes, while direct effects of SDB status failed to predict cognitive findings. These effects were robust when using different characterizations of SDB and were extremely consistent in the context of the methodological differences that are embedded in the various approaches to mediation. In addition, important demographic variables were adjusted for in both mediation and outcome models. To our knowledge, these findings represent the first demonstration of behavior as a mediator of SDB-related effects on cognitive functioning. These results support and extend prior reports that illustrated the presence of a significant relationship between behavior and educational outcomes in children with SDB (23). This suggests that behavior and psychiatric problems that often occur among children with SDB, such as inattention, emotional pathology, or conduct problems, may potentially disrupt natural learning processes that develop and enhance overall cognitive functioning during early formative years.

Because our primary interest was in examining overall cognitive functioning as an outcome, we utilized a strong and divergent set of cognitive functioning measures in these analyses. It is, therefore, worth noting that our measured cognitive domain reflects a variety of reasoning, conceptual, and linguistic abilities that may constitute some of the principal underpinnings of academic achievement to some extent. Although our results suggest that parent-reported behavioral and psychiatric problems fully mediated the relationship between SDB and cognition, this effect, and the potential contribution of behavioral problems toward development of cognitive functioning in this population, could certainly differ based on type of cognitive task involved. Indeed, prior research has suggested that some cognitive measures, such as verbal intelligence and linguistic functioning, may be more sensitive to behavior and attention problems in children due to the experiential and academic nature of the development of these areas during childhood (5, 56). Accordingly, future efforts to examine the potential differences in the behavioral mediation effects based on the type of cognitive task, and whether more linguistic or crystallized aspects of intelligence, and executive aspects of cognitive functioning, are indeed more susceptible to behavioral problems. At present, it remains unknown whether immutable reductions in cognitive functioning follow early childhood neurological and behavioral pathology, or whether a SDB–cognitive phenotype exists. Both scenarios could explain the difficulty in identifying improvements in cognition following treatment for SDB. Recent findings suggesting improvement following treatment in only non-verbal aspects of cognition [e.g., Ref. (13, 57)] may further implicate that behavior and attention problems in early childhood may be the predominant phenotypic antecedent of cognitive deficits. However, it remains entirely plausible that this is primarily true of certain aspects of cognitive functioning and that other aspects are particularly susceptible to the episodic hypercapnia, intermittent hypoxemia, and frequent arousals that occur during childhood SDB.

As would be expected, several methodological issues and limitations are notable and deserve mention. First, our sample was community based but enriched for snoring, such that the possibility exists, albeit remote, that the strength of the models may be susceptible to the recruitment strategy. To this extent, we cannot infer whether such mediation effects would be sustained in a clinically referred population. In addition, other variables of interest could not be examined here; perhaps most notable are socioeconomic status, parental substance abuse, prematurity, and gestational variables. Furthermore, the additional contributions of disrupted sleep microarchitecture originating from periodic leg movements or other sleep disruptors were not specifically investigated and could have further added to the currently uncovered contributions of snoring. The absence of longitudinal follow-up clearly precludes further inferences as to the reversibility of the potential effects outlined here, and is also notable. Despite the aforementioned sensitivity analysis and the plausibility that confounders may be operationally disruptive of the mediation models reported herein, the observational nature of the data requires the use of appropriate caution when making causal assertions based on these analyses.

In summary, this study supports the role of behavioral problems as a mediator of the SDB–cognitive functioning relationships. Our assessments used recently developed sophisticated mediation approaches and the congruence of their findings supports growing evidence showing higher directional sensitivities of behavior–SDB relationships, the role of indirect effects of SDB on cognition through behavior problems and further provides a possible mechanism for the difficulty in detecting improvements in cognitive functioning following treatment for SDB among school-aged children. Thus, early behavior and attention problems may be implicated in lasting, and potentially immutable, cognitive functioning problems unless such issues are detected and intervention occurs very early in the course of their temporal trajectories. Future research should attempt to assess the potential relationships between individual behavioral and attentional variables and specific cognitive outcomes, i.e., the existence of neurobehavioral phenotypic clusters in pediatric SDB, as well as whether specific phenotypes are reversible with treatment of SDB.

## Ethics Statement

This study was carried out in accordance with the recommendations of University of Louisville and University of Chicago medical centers’ Institutional Review Board with written informed consent from all subjects and/or their parents. All gave written informed consent in accordance with the Declaration of Helsinki.

## Author Contributions

DS conceptualized and created the manuscript and conducted analyses, DG and LK-G assisted in conceptualization and manuscript creation and collected participant data, and SH assisted in manuscript creation. DS is the guarantor of this manuscript, and as lead author affirms that the manuscript is an honest, accurate, and transparent account of the study being reported; that no important aspects of the study have been omitted.

## Conflict of Interest Statement

The authors declare that the research was conducted in the absence of any commercial or financial relationships that could be construed as a potential conflict of interest.

## Footnotes

^1^To examine the possibility of cognition serving as a mediator of the SDB–behavior relationship this model was also examined. However, it was found to exhibit a comparatively poor fit to existing data, as expected based on theory driving this research.

^2^Non-parametric (asymptotic distribution-free) analytic methods were also examined, though the pattern of results did not differ appreciably, and were, therefore, not reported here.

^3^Models utilizing factor scores obtained using sum and Bartlett score procedures were also examined, though results did not differ appreciably from FIML estimates, and were therefore not reported here.

## Abbreviations

ADHD, attention deficit hyperactivity disorder; AHI, apnea-hypopnea index; CBCL, child behavior checklist-revised; CPRS-R, Conners’ parent rating scales-revised; HS, habitual snoring; NPSG, nocturnal polysomnogram; RBM, resampling-based mediation; RMPWs, ratio-of-mediator-probability-weights; SDB, sleep-disordered breathing.
